# Biobanking across the phenome - at the center of chronic disease research

**DOI:** 10.1186/1471-2458-13-1094

**Published:** 2013-11-25

**Authors:** Medea Imboden, Nicole M Probst-Hensch

**Affiliations:** 1 Swiss Tropical and Public Health Institute, Basel, Switzerland; 2 University of Basel, Basel, Switzerland

**Keywords:** Comorbidities, Cohort, Genome wide association study, Non-communicable disease, Phenome, Public health, Risk factors

## Abstract

**Background:**

Recognized public health relevant risk factors such as obesity, physical inactivity, smoking or air pollution are common to many non-communicable diseases (NCDs). NCDs cluster and co-morbidities increase in parallel to age. Pleiotropic genes and genetic variants have been identified by genome-wide association studies (GWAS) linking NCD entities hitherto thought to be distant in etiology. These different lines of evidence suggest that NCD disease mechanisms are in part shared.

**Discussion:**

Identification of common exogenous and endogenous risk patterns may promote efficient prevention, an urgent need in the light of the global NCD epidemic. The prerequisite to investigate causal risk patterns including biologic, genetic and environmental factors across different NCDs are well characterized cohorts with associated biobanks. Prospectively collected data and biospecimen from subjects of various age, sociodemographic, and cultural groups, both healthy and affected by one or more NCD, are essential for exploring biologic mechanisms and susceptibilities interlinking different environmental and lifestyle exposures, co-morbidities, as well as cellular senescence and aging. A paradigm shift in the research activities can currently be observed, moving from focused investigations on the effect of a single risk factor on an isolated health outcome to a more comprehensive assessment of risk patterns and a broader phenome approach. Though important methodological and analytical challenges need to be resolved, the ongoing international efforts to establish large-scale population-based biobank cohorts are a critical basis for moving NCD disease etiology forward.

**Summary:**

Future epidemiologic and public health research should aim at sustaining a comprehensive systems view on health and disease. The political and public discussions about the utilitarian aspect of investing in and contributing to cohort and biobank research are essential and are indirectly linked to the achievement of public health programs effectively addressing the global NCD epidemic.

## Background

The aim of the present report is to address the importance of studying non-communicable diseases (NCDs) and their relationship to aging in a systems approach. Understanding the complexity and interrelation of risk factors and disease networks requires the biologic sample collection, detailed and comprehensive phenotyping, and broad risk factor data. We present the international progress made in establishing large population-based biobank cohorts with the explicit aim to investigate non-communicable disease (NCD) etiology longitudinally. We point to the current inadequacy and the critical need to invest substantial research funding into NCD research in low and middle income countries in which the rise of NCDs converges with the high prevalence of infectious diseases. We discuss the relevance of studying pathophysiologic mechanisms linking different age-related NCDs and the aging process. We also highlight recent examples of phenome approaches. Finally, we point out striking pleiotropic findings of NCD phenotypic traits and genome-wide associations (GWAS) which clearly signpost an on-going paradigm shift in NCD research and underscore the potential of agnostic, complex data, systemic and multi-levelled methodologies leading to new understanding of chronic disease etiology.

### International trend for prospective large-sized biobank cohorts

NCDs convey more than 50% of the global burden of disease and are challenging the health of populations worldwide. In high income countries (HICs), the epidemic of NCDs has been recognizably the major public health challenge over the last decades [1]. For this reason several HICs have increased their research efforts and invested substantial funding in extremely large population-based prospective cohort studies (with samples sizes over 200′000). These mega-cohorts (Table 1; see also http://www.p3g.org) apply detailed phenotype descriptions over time, exhaustive temporal assessment of personal and environmental information and include high quality biologic sample collections for future genetic and functional analyses [2]. Prospective biobanking represents a powerful tool for establishing causal relationships as the time-order of sampling and of phenotyping is generally clear. Both hypothesis-driven as well as agnostic research can be conducted. Biologic samples in research can be used to test genetic determinants (e.g. genetic variants of N-Acetyltransferases, *NAT1* and *NAT2*) potentially mediating susceptibility (e.g. increased cancer risk), to discover or validate biomarkers as land marks of mechanisms (e.g. acetylation of aromatic and heterocyclic amines), or to sense and estimate individual environmental exposures (e.g. variable toxicity of carcinogens). Additional applications are expected to increase in the future. General good practices for biobanking in research have been defined (e.g. http://www.ieaweb.org for epidemiologic settings). An increasing need for biologic samples has therefore been the driving force to establish biobank collections in various clinical and observational settings [3]. A well-known example is the UK biobank, collecting blood, saliva and urine of more than 500′000 participants. Questionnaire and measurement data were collected at baseline and follow-up examinations are performed in subsets of the cohort [4]. There are also efforts of similar dimension in low and middle income countries (LMIC) such as the Chinese Biobank Study [Kadoorie Study of Chronic Disease in China (KSCDC)]. This project is a blood-based health database aiming at collecting genetic, environmental and lifestyle data on 510′000 adults aged 30–79 years to understand the causes, risk factors, pathogenesis, prevalence patterns and trends of major infectious and NCDs [5]. The establishment, maintenance and repeated collection of participant data require a substantial long-term investment of research funds. Critical voices point to the tremendous costs and to the methodological challenges to keep bias low over a long follow-up time in a multi-centric study design. But the scientific utility of such large longitudinal datasets is undisputed [6,7]. Understanding the genetic, molecular and mechanistic background of interdependence between NCDs, comorbidities and risk factors during the aging process is a research priority for public health. Sufficiently powered cohorts and biobanks with broad, yet refined characterization of participants for risk factors and health phenotypes are a *conditio sine qua non* to achieve this goal.

**Table 1:** Selection of ongoing mega-cohort studies in adults

**Web site**	**Cohort study**	**Country**	**Country size**	**Focus**	**Sample size**	**Baseline**	**Biologic samples**
--	CONOR/HUNT	Norway	4,9 Mio	Common disease etiology	185′000	1994-1995	Blood
http://www.millionwomenstudy.org/	Million women study	United Kingdom	62,3 Mio	Women’s health	1′300′000	1996 - 2001	Blood, saliva, in a sub-sample
http://epic.iarc.fr/	EPIC	Europe	738,2 Mio	Nutrition, life style and cancer other diseases	520′000	1997	Blood
--	Mexico city prospective study	Mexico	117,4 Mio	Major determinants of morbidity and premature mortality	150′000	1998-2004	Blood
http://www.decode.com/research/	deCODE	Iceland	0.4 Mio	Research company	200′000	2000	Various
http://www.milleniumcohort.org/	Millennium	USA	313,3 Mio	US military family cohort	150′000	2001	Not specified
http://www.geenivaramu.ee/en/	Estonian biobank	Estonia	1.3 Mio	Biologic resource	50′000	2002	Blood
http://www.birmingham.ac.uk/research/activity/mds/projects/HaPS/PHEB/Guangzhou/index.aspx	Guanghzou Biobank Cohort study	China	1339,7 Mio	Genetic, lifestyle, occupational and environmental factors, and life course Causes of the common chronic diseases	40′000	2003	Blood, urine
http://www.ckbiobank.org/	China Kandoorie Biobank	China	1339,7 Mio	Chronic disease etiology, complex interplay of lifestyle, environmental, and genetic susceptibility	500′000	2004-2008	Blood
http://www.phri.ca/pure/index.html	PURE	Several countries	3223,7 Mio	Maladaptation to urbanization and cardiovascular health	120′000	2006	Blood
http://www.ukbiobank.ac.uk/	UK biobank	United Kingdom	62,3 Mio	Common disease etiology	500′000	2007 - 2010	Blood, saliva, urine
http://www.lifelines.net/	LifeLines	The Netherlands	16,8 Mio	causes and prognosis of burden of disease, co-determinants, rather than comorbidity, family study	165′000	2007	Blood, urine
https://www.etude-nutrinet-sante.fr/fr/common/login.aspx	Nutrinet Santé	France	65,4 Mio	Nutrition and health	500′000	2009	Blood, urine
http://www.partnershipfortomorrow.ca/	The Canadian Partnership for Tomorrow Project (CPTP)	Canada	34,4 Mio	Cancer and chronic disease etiology	300′000	2009	Blood
http://lifegene.ki.se/	Life gene	Sweden	9,4 Mio	Nealth and lifestyle	500′000	2011	Blood, urine
http://www.constances.fr	CONSTANCES	France	65,4 Mio	Biologic and research resource	500′000	2011	Blood
http://www.nationale-kohorte.de/index_en.html	German national cohort	Germany	81,8 Mio	Common disease etiology	200′000	2012	Blood

Listed by date of baseline examination start.

## Discussion

### Biobank cohorts and chronic disease research in low and middle income countries (LMIC)

Research on NCDs almost exclusively relies on cohort data and associated biological samples collected in HICs. The recent update of global burden of disease estimates marks a shift from communicable towards non-communicable diseases and from life years lost due to premature death to increased number of years lived with chronic diseases and disabilities in LMICs [1,8]. Though regionally heterogeneous, the LMICs show a persistently high proportion of infectious diseases in addition to a recent increase in prevalence of NCDs such as ischemic heart disease, stroke and diabetes. This observed convergence of NCDs and communicable diseases causes a dual burden of disease [9] for which most LMICs not only lack adequate health system resources, but also research funds to address the regional and local public health challenges [10]. Though causal relationships of NCD etiology and preventive measures identified in population-based biobank cohort studies in HICs will most likely inform public health decisions in LMICs, it is obvious that repeating studies of established NCD risk factors in LMICs will be necessary for proper estimation of their contribution to the disease burden [11]. Much can be learned about effect modifiers and risk factors by paralleled establishment of biobank cohorts in different settings. From human genome variation studies we already know that many African populations harbour a larger degree of genetic variation [12]. Several examples of high quality cohort study efforts in LMIC have been undertaken [7] (Table 1). For example, the prevalence of healthy lifestyle in patients with cardiovascular disease (n = 7519) was investigated in the PURE study, a large-scale epidemiological study that recruited >140,000 individuals residing in in 17 low-, middle-, and high-income countries around the world, and revealed strong correlation of decreasing levels of healthy lifestyle with decreasing country income level [13]. The Guangzhou Biobank Cohort Study [14], combining the use of biomarkers and questionnaire data for investigation of NCDs health system use as well as NCDs etiology, is another excellent example of a regional population-based cohort study in a country transiting fast from low to high income settings, albeit with large social discrepancies. Such large scale biobank cohort studies in LMICs face numerous challenges including funding, political, cultural and religious issues, but they are imminently important to collect data and monitor the dynamics of changes in environmental, life style, societal and health parameters with the increasing trend of urbanization in these countries [7]. They also contribute importantly to increasing the global competitiveness of research in LMICs [15].

### Phenome approach towards disease networks

In aiming to improve understanding of NCD etiology refined phenotyping of specific health outcomes is a necessity. Clinical disease diagnosis based research is known to be challenged by phenotypic heterogeneity. As an example, asthma, an intermittent chronic respiratory disease can be defined as a clinical diagnosis of asthma, but it is known that there are important differences in etiology and mechanisms depending on age of asthma onset or the presence of atopy and allergies. GWAS findings clearly revealed that the locus 17q21 determined childhood and not adult onset asthma [16,17]. Statistical clustering approaches applied to the multilayer disease characteristics of a large group of asthmatic patients identified four distinct asthma phenotype groups: active treated allergic childhood-onset asthma; active treated adult-onset asthma; inactive or mild untreated asthma differing by atopy status and age of asthma onset [18]. In general up to recently, genetic investigations of NCD determinants, especially in large-scale GWAS meta-analyses, reduced the phenotype studied to a clinical diagnostic entity, a fact that may contribute to the disappointingly low predictive power of common genetic disease variants identified to date [19-21]. The importance of precise phenotyping for identifying the genetic contribution to common disease has been stressed since the time point of completion of the human genome project [22]. Clearly this challenges meta-analyses of data from different medium-sized cohorts collected in non-harmonized ways. International efforts to develop harmonized phenotype definitions lead early on to the Human **Phenome** Project [22,23]. Since the initiative call phenome based databases were established (e.g. bipolar disorder phenome [24]; epilepsy phenome/genome project [25]; mouse phenome [26]; human pathology centered phenomes on cardiomyopathy [27], deafness [28], cardiac conduction characteristics [29], human skeletal phenome [30]). Phenotypes forming the basis of the phenome approaches can refer to any characteristic or trait measureable in an organism. It can be as diverse as a morphologic, biochemical, physiological, electrical, behavioral, epigenetic trait and these measures show a large inter-individual variability. Recently phenome-based approaches proofed their usefulness in identifying context-dependent clinical reference values for white blood cell counts [31]. Other recent phenome approaches applied semantic web technologies to scan electronic health records comprising clinical and biologic medical data for identifying genotype-phenotype associations [32,33]. The current applications of the phenome approaches illustrate well the broad definition of the “phenome” summarizing often a large collection of phenotypes. Refined phenome approaches must be expanded to the concept of disease networks [34,35], the **Diseasome**. According to a European population-based survey 25% of the respondents of age older than 14 years reported the presence of more than one chronic condition [36]. A systematic evidence review reported prevalence ranges of multi-morbidity in elderly of 55% to 98% [37]. The identification and clustering of human disease etiologic factors was undertaken in a bioinformatic driven data-mining approach using MeSH annotation of MEDLINE-referenced articles and the authors produced the etiome profile for 863 diseases (available at http://etiome.stanford.edu) [38]. New analytical approaches open novel exploratory avenues of investigation supporting the paradigm shift towards systematic, multi-layered and more exhaustive phenotypic catalogs. Patient records from a 1.5 million large patient population were used to establish correlation links of 161 disorders with disease phenotypes allowing to estimate the genetic overlap within the disease network [39]. A comorbidity database, the human disease network, was established from the analysis of 30 Mio Medicare patient data linking diseases and comorbidities (available at http://hudine.neu.edu/) [40]. More recently, to better understand disease similarities independent research groups have explored the clustering of genome-phenome correlations on a large number of published phenotype – gene associations [41], or the type 2 diabetes genetic loci [42] or the major histocompatibility complex class II surface receptor, HLA-DRB1 [43]. These reports clearly proof the huge potential of bioinformatics-driven data-mining methodologies to shape the diseasome by classification of disease phenotypes and molecular diseases pathways. Thus such public health relevant research will continue to steadily improve our understanding of the phenotypic overlap of different NCDs and their link to aging processes. These system approaches to disease must furthermore be paralleled by systems approaches to understand risk factors. The concept of the phenome has thus been supplemented by the concept of the **Exposome** which measures environmental exposure as internal intermediate phenotypes of exposed organisms [44-46] using metabolomic and proteomic methods for quantification of molecular traits.

### Accelerated aging processes as a link to NCD comorbidity

Given that NCDs are chronic the proportion of comorbidities or secondary NCDs increase with age. Beyond this play of chance, NCD risk factors are known to accelerate the aging process of various organs. Smoking and obesity are among the most consistent factors showing adverse effects on all features of aging. For example, smoking, a potent risk factor for cardiovascular and respiratory NCDs has been suggested to promote cellular senescence of the lung [47], to impair the immune response [48] and increase skin aging [49]. Likewise obesity, a major risk factor for cardiovascular NCDs has been associated with age-related disease of the CNS [50]. Telomere shortening, a marker of the aging process, is inversely associated with several risk factors of diabetes and mitochondrial function in diabetic patients compared to healthy controls [51]. Telomere length was positively correlated with good glycemic/lipid control and negatively correlated with adiposity and insulin resistance [51,52]. Other NCD risk factors such as sun light or weight loss exhibit adverse effects on more restricted features of aging such as skin aging or osteoporosis (see Table 2 as illustrative example).

**Table 2:** Risk factors of NCDs and aging

**Risk factor studied**	**Disease or trait**	**Acclerated aging and impaired function**	**Reference**
Smoking	Humoral immunity	Immune system aging	[48]
	Inflammatory response	Immune system aging	[53]
	Heart rate variabiltiy	Autnomous nervous system aging	[54]
	Alzheimer	Premature cognitive impairment, CNS aging	[55]
	Atherosclerosis	Cardiovascular aging	[56]
	Elastosis of the neck	Skin aging	[57]
	Bone mineral density	Bone aging	[58]
Obesity, BMI, high calorie intake,	Impaired immune response	Immune system aging	[59]
Waist-hip ratio, skin-folds,	CD8 Tcell activation	Immune system aging	[59]
Body weight	Lipodystrophy	Adipocyte aging	[60]
	Heart rate variabiltiy	Autnomous nervous system aging	[61]
	Alzheimer	Premature cognitive impairment, CNS aging	[55]
	Atherosclerosis	Cardiovascular aging	[56]
	Alopecia	Hair aging	[62]
	Bone mineral density	Bone aging	[58]
Dyslipidemia	Atherosclerosis	Cardiovascular aging	[56]
	Alopecia	Hair aging	[62]
History of diabetes	Alzheimer	Premature cognitive impairment, CNS aging	[55]
	Bone mineral density	Bone aging	[58]
Hypertension,	Alzheimer	Premature cognitive impairment, CNS aging	[55]
High resting pulse	Atherosclerosis	Cardiovascular aging	[56]
	Osteoporosis	Bone aging	[63]
	Bone mineral density	Bone aging	[58]
Other chronic diseases,	Immunosenescence	Immune system aging	[64]
Comorbidity	Lipodystrophy	Adipocyte aging	[65]
	Atherosclerosis	Cardiovascular aging	[56]
	Sacropenia	Muscle aging	[66]
	Osteoporosis	Bone aging	[63]
Medication intake	Sacropenia	Muscle aging	[66]
	Osteoporosis	Bone aging	[63]
UV light/sun exposure	Alopecia	Hair aging	[62]
Low sun exposure	Elastosis of the neck	Skin aging	[57]
	Sacropenia	Muscle aging	[66]
Health behaviours	Alzheimer	Premature cognitive impairment, CNS aging	[55]
Low level of mental activity	Atherosclerosis	Cardiovascular aging	[56]
Physical inactivity	Sacropenia	Muscle aging	[66]
	Osteoporosis	Bone aging	[63]
Depression	Atherosclerosis	Cardiovascular aging	[56]
Poor diet	Sacropenia	Muscle aging	[66]
Weight loss/no weight gain	Osteoporosis	Bone aging	[63]
Low education	Alzheimer	Premature cognitive impairment, CNS aging	[55]
	Atherosclerosis	Cardiovascular aging	[56]
Psychosocial factors	Alzheimer	Premature cognitive impairment, CNS aging	[55]

Content of table is illustrative, not exhaustive.

The natural history of aging is characterized by a diminished self-renewal capacity of the organism resulting in sclerodermatous changes of the skin, alopecia, osteoporosis, sarcopenia, muscle atrophy, generalized lipodystrophy, atherosclerosis, decreased elasticity of the vascular system, immunologic senescent changes such as decline in humoral immunity, T-cell functional dysregulation, innate and adaptive immune functions [48,59,64,67]. Characteristic land marks of aging are also neurologic senescent changes of the central, peripheral and autonomic nervous system including limited neuronal loss, glial proliferation in the cortex and an overall brain weight decrease, degradation of sensory performance, decline in proprioception and somatosensory information processing and also reduced reactivity of the sympathetic and the parasympathetic nervous activity [68,69]. It is likely that systemic approaches combining the focus on accelerated aging, NCDs, environmental and genetic risk factors will point to the underlying disease biology. Understanding how shared risk factors affect mechanisms common to NCDs and aging processes is important from a public health perspective to meet effective prevention programs.

### Lessons learned from genetics on NCD clustering: pleiotropic gene variants

Despite ongoing debates about the limitation of GWAS findings from the predictive personalized medicine perspective, GWAS studies do not announce the end of complex disease genetics, but rather a promising first step. Completely novel genes expand our understanding of NCD pathology. A large number of GWAS loci have been consistently associated with one or multiple NCDs in independent populations (see Additional file 1; http://www.genome.gov/gwastudies). Evidence for pleiotropy of loci, genes and even specific SNPs suggests important mechanistic links between diseases and is of potential relevance to advance understanding the biology of NCD clusters, co-morbidities and aging processes. A recent meta-analysis of 372 GWAS on 105 unique age-related diseases revealed the clustering of genetic variants in ten significantly enriched chromosomal locations which contain genes involved in inflammation and cellular senescence [70]. Pleiotropy is defined as a genetic variant or a gene having an effect on multiple phenotypes. In Table 3, we present an overview of specific SNPs likely to be pleiotropic. They were consistently associated with different forms of cancer (i.e. **rs401681**, *TERT*, *CLPTM1L*, 5p15.33 – associated with lung, bladder, pancreatic cancer, melanoma and prostate-specific antigen levels) and of chronic inflammatory diseases (i.e. **rs11209026**, *IL23R*, 1p31.3 – associated with Crohn’s disease, ulcerative colitis, ankylosing spondylitis and psoriasis; **rs10488631**, *IRF5,TNPO3*, 7q32.1 – associated with systemic lupus erythematosus, systemic sclerosis, rheumatoid arthritis and primary biliary cirrhosis; see Additional file 2 for detailed summary of pleiotropic SNPs). This observed non-random clustering of NCD-linked traits and specific pleiotropic SNPs can be used to identify biologic mechanisms shared by different NCDs. In a recent study a method was presented to evaluate the pleiotropy among GWAS-identified SNPs and genes for common complex disease and traits; it reported that 17% of the GWAS genes and 4% of the GWAS SNPs showed evidence of pleiotropy [71]. Although pleiotropy had been suggested to be common to the genetic architecture of complex disease [72], only isolated cases of pleiotropy had been reported previously such as the links between *APOE* genotypes and dyslipidemia, coronary heart disease and Alzheimer's disease [73], and type 2 diabetes and prostate cancer (*TCF2 genotypes*) [74]. The genetic overlap between psoriasis, diabetes type 2 and Crohn’s disease, three inflammatory diseases affecting distinct organs, was identified by combining evidence from linkage and GWAS data [75]. Recently antagonistic pleiotropic effects of genetic variants were evidenced conferring risk for one disease, diabetes type 1, and protection for another disease, inflammatory bowel disease [76].

**Table 3:** Pleiotropic GWAS loci of NCDs

**Locus, gene**	**dbSNP ID**	**NCD entity associated with SNP**	**P-value**	**Risk allele frequency**	**PubMed ID**
**Cancer linked NCDs cluster**					
5p15.33, TERT	rs2736100	Glioma	2.00E-17	0.49	19578367
	rs2736100	Glioma	1.00E-14	NR	21531791
	rs2736100	Glioma	7.00E-09	NR	21827660
	rs2736100	Hematological and biochemical traits	3.00E-08	0.4	20139978
	rs2736100	Idiopathic pulmonary fibrosis	3.00E-08	0.41	18835860
	rs2736100	Lung adenocarcinoma	2.00E-22	0.39	20700438
	rs2736100	Lung adenocarcinoma	3.00E-11	0.39	20871597
	rs2736100	Lung cancer	1.00E-27	0.41	21725308
	rs2736100	Testicular germ cell cancer	8.00E-15	0.49	20543847
5p15.33, TERT, CLPTM1L	rs401681	Bladder cancer	5.00E-07	0.54	20972438
	rs401681	Lung cancer	8.00E-09	NR	18978787
	rs401681	Melanoma	3.00E-08	0.46	21983787
	rs401681	Pancreatic cancer	7.00E-07	0.45	20101243
	rs401681	Serum prostate-specific antigen levels	1.00E-10	0.55	21160077
8q24.21, Intergenic	rs6983267	Colorectal cancer	1.00E-14	0.49	17618284
	rs6983267	Colorectal cancer	7.00E-11	0.48	18372905
	rs6983267	Colorectal cancer	2.00E-08	0.34	21242260
	rs6983267	Prostate cancer	9.00E-13	0.5	17401363
	rs6983267	Prostate cancer	9.00E-13	0.49	18264097
	rs6983267	Prostate cancer	7.00E-12	0.53	18264096
	rs6983267	Prostate cancer	9.00E-06	NR	21743057
9p21.3, CDKN2A, CDKN2B	rs4977756	Glaucoma	1.00E-14	0.6	21532571
	rs4977756	Glioma	7.00E-15	0.6	19578367
**Inflammatory trait linked NCDs cluster**					
1p31.3, IL23R	rs11209026	Ankylosing spondylitis	2.00E-17	0.93	21743469
	rs11209026	Ankylosing spondylitis	9.00E-14	0.94	20062062
	rs11209026	Crohn’s disease	1.00E-64	0.93	21102463
	rs11209026	Crohn’s disease	4.00E-21	NR	22293688
	rs11209026	Crohn’s disease	2.00E-18	0.92	17447842
	rs11209026	Inflammatory bowel disease	4.00E-11	0.93	17068223
	rs11209026	Inflammatory bowel disease	7.00E-11	0.94	18758464
	rs11209026	Psoriasis	7.00E-07	NR	20953190
	rs11209026	Ulcerative colitis	5.00E-28	0.94	21297633
	rs11209026	Ulcerative colitis	3.00E-10	NR	19915572
	rs11209026	Ulcerative colitis	1.00E-08	0.93	19122664
1p13.2, PTPN22	rs2476601	Crohn’s disease	1.00E-08	0.9	18587394
	rs2476601	Rheumatoid arthritis	9.00E-74	0.1	20453842
	rs2476601	Rheumatoid arthritis	2.00E-21	NR	19503088
	rs2476601	Rheumatoid arthritis	2.00E-11	0.1	17804836
	rs2476601	Type 1 diabetes	9.00E-85	NR	19430480
	rs2476601	Type 1 diabetes	2.00E-80	0.09	17554260
	rs2476601	Type 1 diabetes	1.00E-07	0.09	17632545
	rs2476601	Type 1 diabetes autoantibodies	2.00E-111	NR	21829393
	rs2476601	Vitiligo	1.00E-07	0.1	20410501
7q32.1, IRF5,TNPO3	rs10488631	Primary biliary cirrhosis	3.00E-10	0.11	20639880
	rs10488631	Primary biliary cirrhosis	2.00E-07	NR	19458352
	rs10488631	Rheumatoid arthritis	4.00E-11	0.11	20453842
	rs10488631	Systemic lupus erythematosus	7.00E-18	0.11	21408207
	rs10488631	Systemic lupus erythematosus	2.00E-11	0.12	18204098
	rs10488631	Systemic sclerosis	2.00E-13	NR	20383147
	rs10488631	Systemic sclerosis	2.00E-10	NR	21779181
	rs10488631	Systemic sclerosis	2.00E-07	NR	21779181
	rs10488631	Systemic sclerosis	4.00E-07	0.09	21750679
18p11.21, PTPN2	rs2542151	Crohn’s disease	5.00E-17	0.15	18587394
	rs2542151	Crohn’s disease	3.00E-08	0.18	17554261
	rs2542151	Crohn’s disease	2.00E-07	0.16	17554300
	rs2542151	Type 1 diabetes	1.00E-14	0.16	17554260
	rs2542151	Type 1 diabetes	9.00E-08	NR	18978792
	rs2542151	Type 1 diabetes autoantibodies	4.00E-13	NR	21829393
18p11.21, PTPN2	rs1893217	Celiac disease	3.00E-10	0.17	20190752
	rs1893217	Celiac disease and Rheumatoid arthritis	5.00E-12	NR	21383967
	rs1893217	Type 1 diabetes	4.00E-15	NR	19430480
**Cardiovascular trait linked NCDs cluster**					
2p23.3, GCKR	rs1260326	Cardiovascular disease risk factors	2.00E-08	0.4	21943158
	rs1260326	Cholesterol, total	7.00E-27	0.41	20686565
	rs1260326	Chronic kidney disease	3.00E-14	0.41	20383146
	rs1260326	C-reactive protein	5.00E-40	NR	21300955
	rs1260326	Hematological and biochemical traits	4.00E-09	0.44	20139978
	rs1260326	Hypertriglyceridemia	7.00E-09	0.41	20657596
	rs1260326	Liver enzyme levels (gamma-glutamyl transferase)	4.00E-13	0.38	22001757
	rs1260326	Metabolic traits	4.00E-10	0.35	19060910
	rs1260326	Platelet counts	9.00E-10	NR	22139419
	rs1260326	Serum metabolites	3.00E-18	NR	22286219
	rs1260326	Triglycerides	6.00E-133	0.41	20686565
	rs1260326	Triglycerides	2.00E-31	0.45	19060906
	rs1260326	Two-hour glucose challenge	3.00E-10	NR	20081857
	rs1260326	Waist circumference and related phenotypes	4.00E-08	NR	18454146
11q12.2, FADS1, FADS2	rs174547	HDL cholesterol	2.00E-12	0.33	19060906
	rs174547	Lipid metabolism phenotypes	8.00E-262	NR	22286219
	rs174547	Metabolic traits	9.00E-116	0.32	21886157
	rs174547	Phospholipid levels (plasma)	4.00E-154	NR	21829377
	rs174547	Phospholipid levels (plasma)	3.00E-64	NR	21829377
	rs174547	Resting heart rate	2.00E-09	0.33	20639392
	rs174547	Serum metabolites	7.00E-179	0.3	20037589
	rs174547	Triglycerides	2.00E-14	0.33	19060906
11q14.3, MTNR1B	rs1387153	Fasting plasma glucose	2.00E-36	0.29	19060909
	rs1387153	Glycated hemoglobin levels	4.00E-11	0.28	20858683
	rs1387153	Metabolic syndrome (bivariate traits)	2.00E-09	NR	21386085
	rs1387153	Metabolic syndrome (bivariate traits)	8.00E-09	NR	21386085
	rs1387153	Type 2 diabetes	8.00E-15	NR	20581827
12q24.12, ALDH2, BRAP	rs671	Coronary heart disease	2.00E-34	0.23	21971053
	rs671	Drinking behavior	4.00E-211	0.75	21372407
	rs671	Esophageal cancer	3.00E-24	NR	19698717
	rs671	Hematological and biochemical traits	7.00E-10	0.26	20139978
	rs671	Hematological and biochemical traits	5.00E-09	0.26	20139978
	rs671	Intracranial aneurysm	3.00E-06	0.75	22286173
	rs671	Triglycerides	2.00E-06	NR	22171074
16q13, CETP	rs3764261	Age-related macular degeneration	7.00E-09	0.33	21665990
	rs3764261	Age-related macular degeneration	7.00E-07	0.32	20385819
	rs3764261	Cholesterol, total	7.00E-14	0.32	20686565
	rs3764261	HDL cholesterol	2.00E-57	0.31	18193043
	rs3764261	HDL cholesterol	7.00E-29	0.28	19060910
	rs3764261	HDL cholesterol	3.00E-12	0.2	19359809
	rs3764261	HDL cholesterol	7E-380	0.32	20686565
	rs3764261	LDL cholesterol	9.00E-13	0.32	20686565
	rs3764261	Lipid metabolism phenotypes	1.00E-36	NR	22286219
	rs3764261	Metabolic syndrome	1.00E-48	0.36	20694148
	rs3764261	Metabolic syndrome	3.00E-13	NR	21386085
	rs3764261	Triglycerides	1.00E-12	0.45	20686565
	rs3764261	Waist circumference	1.00E-27	NR	18454146
19p13.2, LDLR	rs6511720	Cardiovascular disease risk factors	5.00E-11	0.11	21943158
	rs6511720	Carotid intima media thickness	1.00E-07	NR	21909108
	rs6511720	Cholesterol, total	7.00E-97	0.11	20686565
	rs6511720	LDL cholesterol	4.00E-117	0.11	20686565
	rs6511720	LDL cholesterol	2.00E-51	0.1	18193044
	rs6511720	LDL cholesterol	2.00E-26	0.1	19060906
	rs6511720	LDL cholesterol	4.00E-26	0.9	18193043
	rs6511720	Lp-PLA2 activity and mass	3.00E-11	0.1	22003152
19q13.32, APOE, APOC1	rs4420638	Alzheimer’s disease	2.00E-44	NR	17998437
	rs4420638	Alzheimer’s disease	1.00E-39	NR	17975299
	rs4420638	Alzheimer’s disease (age of onset)	1.00E-12	NR	22005931
	rs4420638	Alzheimer’s disease (late onset)	1.00E-39	NR	17474819
	rs4420638	Cholesterol, total	5.00E-111	0.17	20686565
	rs4420638	Cognitive decline	4.00E-27	NR	22054870
	rs4420638	C-reactive protein	9.00E-139	NR	21300955
	rs4420638	C-reactive protein	5.00E-27	NR	19567438
	rs4420638	C-reactive protein	3.00E-07	0.9	21196492
	rs4420638	HDL cholesterol	4.00E-21	0.17	20686565
	rs4420638	LDL cholesterol	9.00E-147	0.17	20686565
	rs4420638	LDL cholesterol	1.00E-60	0.2	18193044
	rs4420638	LDL cholesterol	3.00E-43	0.18	18193043
	rs4420638	LDL cholesterol	2.00E-40	0.18	20864672
	rs4420638	LDL cholesterol	4.00E-27	0.16	19060906
	rs4420638	LDL cholesterol	1.00E-20	0.18	18262040
	rs4420638	LDL cholesterol	2.00E-07	NR	18802019
	rs4420638	Lp-PLA2 activity and mass	5.00E-30	0.84	22003152
	rs4420638	Lp-PLA2 activity and mass	6.00E-24	0.16	20442857
	rs4420638	Longevity	2.00E-16	0.81	21740922
	rs4420638	Quantitative traits	3.00E-07	0.21	19197348
	rs4420638	Triglycerides	3.00E-13	0.22	17463246
**Cardiovascular & inflammatory trait linked NCDs cluster**					
12q24.12, SH2B3	rs3184504	Coronary heart disease	6.00E-06	0.44	21378990
	rs3184504	Diastolic blood pressure	4.00E-25	0.47	21909115
	rs3184504	Diastolic blood pressure	3.00E-14	0.48	19430479
	rs3184504	Eosinophil counts	7.00E-19	0.38	19198610
	rs3184504	Rheumatoid arthritis	6.00E-06	0.51	20453842
	rs3184504	Systolic blood pressure	5.00E-09	0.48	19430479
	rs3184504	Type 1 diabetes	3.00E-27	NR	19430480
	rs3184504	Type 1 diabetes autoantibodies	2.00E-38	NR	21829393
12q24.12, SH2B3, ATXN2	rs653178	Blood pressure	7.00E-20	0.59	21909110
	rs653178	Celiac disease	7.00E-21	0.5	20190752
	rs653178	Celiac disease	8.00E-08	0.48	18311140
	rs653178	Celiac disease and Rheumatoid arthritis	3.00E-19	NR	21383967
	rs653178	Chronic kidney disease	4.00E-11	0.5	20383146
	rs653178	Diastolic blood pressure	3.00E-18	0.53	19430483
**Skin pigmentation linked NCDs cluster**					
11q14.3, TYR	rs1393350	Blue vs. green eyes	3.00E-12	0.23	17952075
	rs1393350	Eye color	3.00E-09	0.27	20585627
	rs1393350	Melanoma	2.00E-14	0.27	19578364
	rs1393350	Melanoma	2.00E-13	0.28	21983787
	rs1393350	Skin sensitivity to sun	2.00E-06	0.27	17952075
	rs1393350	Tanning	2.00E-13	NR	19340012
	rs1393350	Vitiligo	2.00E-18	0.73	20410501
16q24.3, MC1R	rs1805007	Basal cell carcinoma	4.00E-17	0.07	21700618
	rs1805007	Blond vs. brown hair color	2.00E-13	0.08	17952075
	rs1805007	Freckles	1.00E-96	0.05	17952075
	rs1805007	Red vs non-red hair color	2.00E-142	NR	17952075
	rs1805007	Skin sensitivity to sun	2.00E-55	0.06	17952075

A selection of GWAS identified pleiotropic SNPs implicated in more than one NCD entity are presented here. For a more complete list of pleiotropic loci see Additional file 1. Data has been downloaded (09 March 2012) from the online catalogue of published GWAS available at http://www.genome.gov/gwastudies.

NR, not reported.

Lp-PLA2, lipoprotein-associated Phospholipase A2.

LDLR, Low density lipoprotein receptor.

HDL, High density lipoprotein.

LDLR, Low density lipoprotein.

### Methodological challenge of data mining and of complex systems analysis

The research community is facing unprecedented statistical, data mining and analytical challenges as the next steps ahead are complex interaction studies of genes, other –omics markers, lifestyle, and environment on the phenome. Standard statistical approaches using linear causal relationships have shown to be limited for reproducible association studies on complex phenotypes as well as for two-way interaction analyses. Researchers will need to adapt their current methods by implementing approaches that reflect more closely the dynamics of adaptive biologic systems by taking non-linear and non-proportional relationships into account. Methods of complex system science and chaos theory have been applied to various biologic systems [77] and have been proposed to be applied to human health behavioral changes for public health prevention aims [78]. Fractal dynamics in physiology have shown to be relevant to disease and aging [79], to biologic signals in general [80] and chaotic motifs have been investigated in dynamic behavior of gene regulatory networks [81]. To date we have only started to investigate disease clusters and pleiotropic risk effects in a systematic manner [82,83]. Formal analytical concepts of disease similarities and shared gene networks have been proposed to guide future research for the identification of molecular evidence of comorbidities [84]. Recent novel data mining approaches to combine GWAS findings and phenome data have been proposed to achieve NCD disease gene discovery, phenotype classification [41] and phenome-wide association studies [85] or to improve disease diagnostic procedures [27,86]. Other bioinfomatic approaches combining animal model data of human disease and mammalian phenotype ontologies databases seem to suggest that germline genetic variation might underlie the heterogeneity of comorbidities [87,88].

## Summary

In the present report, we covered a wide range of aspects of importance to NCD research, including establishments and maintenance of large and systematic biobank cohorts from all parts of the world; implementation of broad and detailed phenotyping, as well as broad and detailed risk factor assessment, including aging characteristics; development of novel analytical methods for systemic analysis, addressing networks of diseases, or of personal and environmental risk factors, as well application of agnostic genomic analysis methods. In fact, to meet current and future public health challenges and to improve efficacy of prevention at the individual as well as at the population level, we need answers to the following questions [89-92]: Which are major pathophysiologic pathways mediating the clustering of NCDs? To what degree are biological mechanisms shared between NCDs and normal aging? Do modifiable NCD risk factors act through common mechanisms? Can persons susceptible to common NCD risk patterns and comorbidities be identified?

To address this type of questions with data providing adequate statistical power and using hypothesis driven and explorative as well as agnostic approaches, establishment and maintenance of carefully designed large and comprehensive population-based cohorts with prospective collection of biological samples are a key requirement. Efforts must be further intensified to collaborate across cohorts from different geographic regions in a harmonized fashion, a process already started with remarkable success in P3G [93]. Harmonized and exhaustive phenotype collection is a particular challenge and novel instruments as developed for standardized assessment of multiple chronic diseases etiology [94] must be implemented. The quality management of a sustainable long-term biobank importantly comprises next to legislative, ethical and financial aspects also guaranteed safety of samples, temperature monitoring, traceability and parsimonious use of sample aliquots. Quality management of biological sample collection is particularly important for cohort studies with multi-centric design.

Given that biobank cohorts serve to increase the wellbeing of future generations by indirectly promoting biomedical knowledge and public health, these activities require the development of normative procedures and defined governance [95,96]. There are still issues left to be resolved, such as establishing large biobanks for investigation of future research questions conflicts with the well accepted and widely implemented personal informed consent [97]. In the light of biobanking’s interest for present and future society, it might be considered a great good [95] and according discussions for a possibility of general non-personalized consent in politics and public are needed. This debate paper aimed to highlight the potential of biobank cohort research for complex disease etiology, a field of research that will allow improving health of populations as well as informing individuals on quality-of life increasing health decisions.

## Competing interests

The authors declare that they have no competing interests.

## Authors’ contributions

Both authors made substantial contributions to the conception and design of the paper. NPH is the principal investigator of the SAPALDIA biobank cohort. MI contributed significantly to the set-up of the SAPALDIA biobank and summarized current database and literature findings. Both authors have been involved in interpreting the data, as well as drafting and critically revising the debate manuscript. MI and NPH have given final approval of the version to be published.

## Pre-publication history

The pre-publication history for this paper can be accessed here:

http://www.biomedcentral.com/1471-2458/13/1094/prepub

## Supplementary Material

Additional file 1 Evidence for pleiotropic loci identified by GWAS. Click here for file

Additional file 2 Evidence for non-random clustering of NCD-linked traits and specific pleiotropic GWAS-identified SNPs. Click here for file
